# Influence of Acidic pH on Hydrogen and Acetate Production by an Electrosynthetic Microbiome

**DOI:** 10.1371/journal.pone.0109935

**Published:** 2014-10-15

**Authors:** Edward V. LaBelle, Christopher W. Marshall, Jack A. Gilbert, Harold D. May

**Affiliations:** 1 Department of Microbiology & Immunology, Marine Biomedicine & Environmental Science Center, Hollings Marine Laboratory, Medical University of South Carolina, Charleston, South Carolina, United States of America; 2 Institute for Genomic and Systems Biology, Argonne National Laboratory, Argonne, Illinois, United States of America; 3 Department of Ecology and Evolution, University of Chicago, Chicago, Illinois, United States of America; 4 Marine Biological Laboratory, Woods Hole, Massachusetts, United States of America; 5 College of Environmental and Resource Sciences, Zhejiang University, Hangzhou, China; Louisiana State University and A & M College, United States of America

## Abstract

Production of hydrogen and organic compounds by an electrosynthetic microbiome using electrodes and carbon dioxide as sole electron donor and carbon source, respectively, was examined after exposure to acidic pH (∼5). Hydrogen production by biocathodes poised at −600 mV vs. SHE increased>100-fold and acetate production ceased at acidic pH, but ∼5–15 mM (catholyte volume)/day acetate and>1,000 mM/day hydrogen were attained at pH ∼6.5 following repeated exposure to acidic pH. Cyclic voltammetry revealed a 250 mV decrease in hydrogen overpotential and a maximum current density of 12.2 mA/cm^2^ at −765 mV (0.065 mA/cm^2^ sterile control at −800 mV) by the *Acetobacterium*-dominated community. Supplying −800 mV to the microbiome after repeated exposure to acidic pH resulted in up to 2.6 kg/m^3^/day hydrogen (≈2.6 gallons gasoline equivalent), 0.7 kg/m^3^/day formate, and 3.1 kg/m^3^/day acetate ( = 4.7 kg CO_2_ captured).

## Introduction

The ability to capture carbon dioxide (CO_2_) from waste streams and convert it into value added chemicals and fuels has attracted increasing interest due to concerns over fossil fuel supply, price, and climate change. Microbial electrosynthesis is a process by which microbes grow as a biocathode and couple electrical energy to the capture and conversion of CO_2_ into compounds such as methane or organic acids [1]–[8]. Ideally the electrical energy would be renewable and sustainable, but it may come from the existing grid during off-peak hours to recover otherwise lost fossil-based energy. Depending on the source of electricity and how the upgraded carbon product is used, the process would then be carbon neutral or negative.

This nascent technology may prove to be more efficient than photosynthetic biomass-based fuel and chemical production in a number of ways. Microbial electrosynthesis is projected to utilize less water and land resources than photosynthetic-based processes, as well as less auxiliary energy inputs such as biomass collection and preprocessing [9]. The electron recovery or coulombic efficiency for total products of microbial electrosynthesis has exceeded 80% [2], [3]. If the process were to receive electrical energy from solar panels (conversion efficiencies ∼20%) then it would best the solar conversion by photosynthetic C4 plants (∼1% in practice, near 6% under optimal conditions); making microbial electrosynthesis an attractive solution for carbon capture and chemical production [10]. Anaerobic acetogens thus far are associated with or capable of performing electrosynthesis, and their metabolism may contribute to the observed efficiency. For example, anaerobic processes have the potential to divert more electrons and carbon into the product (>90%) than aerobic processes (<10%), as well as reducing the exposure to degradative and toxic reactive oxygen species [11]. The ancient carbon fixing pathway of acetogens, the Wood-Ljungdahl pathway, is more efficient than other carbon fixation pathways, requiring only 8 enzymes, less than one mole of ATP and just over 4 moles of hydrogen per mole of acetyl-CoA produced [12]. The efficient catalysis of carbon-carbon bond formation under ambient conditions makes this an attractive carbon fixation pathway to transform electrons into chemicals, fuels, and polymers at high yields.

Electrosynthesis of acetate and other short-chain fatty acids from CO_2_ has been examined with media (catholyte) buffered at near neutral pH [1]–[6], and an electrosynthetic microbiome was maintained in batch with a cathode poised at −590 mV vs. SHE [1], [2]. Regular exchanges of the catholyte under a constant supply of CO_2_ will maintain the pH close to neutrality and thereby support acetogenesis [1], [2]. However, little is known about the response of the electrosynthetic microbiome to acidic pH. Here we show that lower pH results in a dramatic increase in H_2_ production by the microbiome, which has implications for H_2_ and organic acid production. This extends our understanding of how these microbiomes perform electrosynthesis, and suggests a platform for the electrosynthetic production of fuels and chemicals [13]–[16].

## Materials and Methods

### Microbial Culture and Media

An electrosynthetic microbiome enriched from brewery wastewater obtained from Palmetto Brewery (Charleston, SC) was used in this study [2]. A phosphate-buffered medium was made using 10.71 g/L dipotassium hydrogen phosphate, 5.24 g/L potassium dihydrogen phosphate, 0.25 g/L ammonium chloride, 0.6 g/L sodium dihydrogen phosphate monohydrate, 0.1 g/L potassium chloride, 0.212 g/L magnesium chloride hexahydrate, 30.4 mg/L calcium chloride dihydrate, and the same mineral and vitamin concentrations reported in Marshall et al. [2]. A bicarbonate-buffered medium was obtained by replacing the potassium phosphate buffer system with 2.5 g/L sodium bicarbonate. Where noted, 50 mM of sodium bromoethanesulfonate was used as a methanogenic inhibitor, or replaced with 50 mM sodium chloride.

### Bioelectrochemical Analysis

Reactors were customized three-electrode, two-chamber glass cells (ChemGlass) separated by a 2 cm^2^ CMI-7000 cation exchange membrane (Membranes International). A total of 25 grams of graphite granules (Showa Denko) were used in each chamber for the anode and cathode. A 0.95 cm outer diameter fine extruded graphite rod (Graphite Store) was cut into 3 cm long current collectors wound with 0.81 mm diameter titanium wire (Sigma Aldrich). All carbon electrodes were pretreated by washing in acetone and drying, followed by immersion in 1 M NaOH, and 1 M HCl for 24 hours each with deionized water rinses between each step. Reference electrodes were made with a 1 mm diameter AgCl coated silver wire (SurePure Metals) immersed in 4 mm glass capillary tube (ChemGlass) containing 3 M KCl saturated with Ag/AgCl and to which a Vycor tip (Koslow) was attached using Teflon heat-shrink tape (BASi). The reference electrodes were immersed in a 7 mm diameter Luggin capillary containing 1 M KCl. The cathode and anode chambers were each filled with 50 mL of media. Reactors were poised at −600 mV vs. the standard hydrogen electrode (SHE) unless indicated otherwise. Chronoamperometry and cyclic voltammetry were recorded using a VMP3 potentiostat and EC-Lab Software (Bio-Logic Science Instruments). Voltammetric sweeps ranged from −800 to 0 mV vs. SHE at 1 mV/sec. Reactors were sparged with 100% carbon dioxide at 15 mL/min, except in graphite rod cathode yield tests. Another larger customized reactor (Adams & Chittenden) was designed with a 20 cm^2^ cation exchange membrane. The anode and cathode chambers each contained 100 g graphite granules and 100 mL of phosphate buffered media. This reactor was used to test the biocathodes at potentials lower than −600 mV vs. SHE. A total of 10 reactors were examined and the inoculation scheme is depicted in Fig. S1.

### Chemical Analysis

Aliquots of media were filtered and analyzed for pH using a pH meter (Mettler-Toledo) and fatty acid content via HPLC (Shimadzu) using the method described in Marshall et al. [2]. A 100 µL glass syringe (Hamilton) was used to sample headspace gas composition via gas chromatography (HP) using the method reported in Marshall et al. [2]. Gas production rate was calculated from the partial pressure and flow rate measured using a flow meter (J&W Scientific). Note that for sparging experiments, hydrogen production is reported as mM/day, while for sealed headspace tests hydrogen accumulation is reported as mM. Maximum production rates refer to within the time course of one batch between media exchanges.

### Scanning Electron Microscopy Analysis of Electrode

Samples for SEM were incubated in a 0.1 M sodium cacodylate buffer with 2% gluteraldehyde for 3 hours, then incubated in a 2.5% osmium tetroxide, and finally dehydrated with an ethanol dilution series using 0%, 25%, 50%, 75%, % and 100% at 5 minute intervals. Samples were stored in a desiccator before being sputtered with Au and Pd using a Denton Vacuum sputter coater. Images were mounted on a stage with conductive carbon tape and imaged using a JEOL JSM-5600LV Scanning Electron Microscope.

### 16S rRNA/DNA Sequencing of Planktonic Community and Cells Attached to Cathode Surface

Reactor 4 was analyzed for microbial composition at 5 months after inoculation. Electrode attached cells were sampled by collecting graphite granules while planktonic cells were collected by filtering supernatant through a Sterivex filter (Millipore). Both samples were stored in Soil Preservation Solution (MoBio Laboratories) at −80°C. DNA from electrode-attached cells was extracted using a PowerSoil DNA Isolation Kit (MoBio Laboratories). DNA from planktonic cells was extracted using PowerWater Sterivex DNA Isolation Kit (MoBio Laboratories). Primers amplifying the V4 region (F515/R806) of 16S ribosomal sequence was amplified using Golay barcoded primers [17], and sequenced on the MiSeq platform (Illumina). 16S analysis was completed with the QIIME v1.7 toolkit. All extraction, amplification, and sequencing methods followed the standards of the Earth Microbiome Project ([18], http://www.earthmicrobiome.org/). Sequences are publicly available under the MG-RAST IDs: Reactor 4 electrode 4562455.3, and Reactor 4 supernatant 4562456.3 ([19], http://metagenomics.anl.gov/).

## Results and Discussion

### Response of the microbiome to a reduction in pH

**Figure 1:**
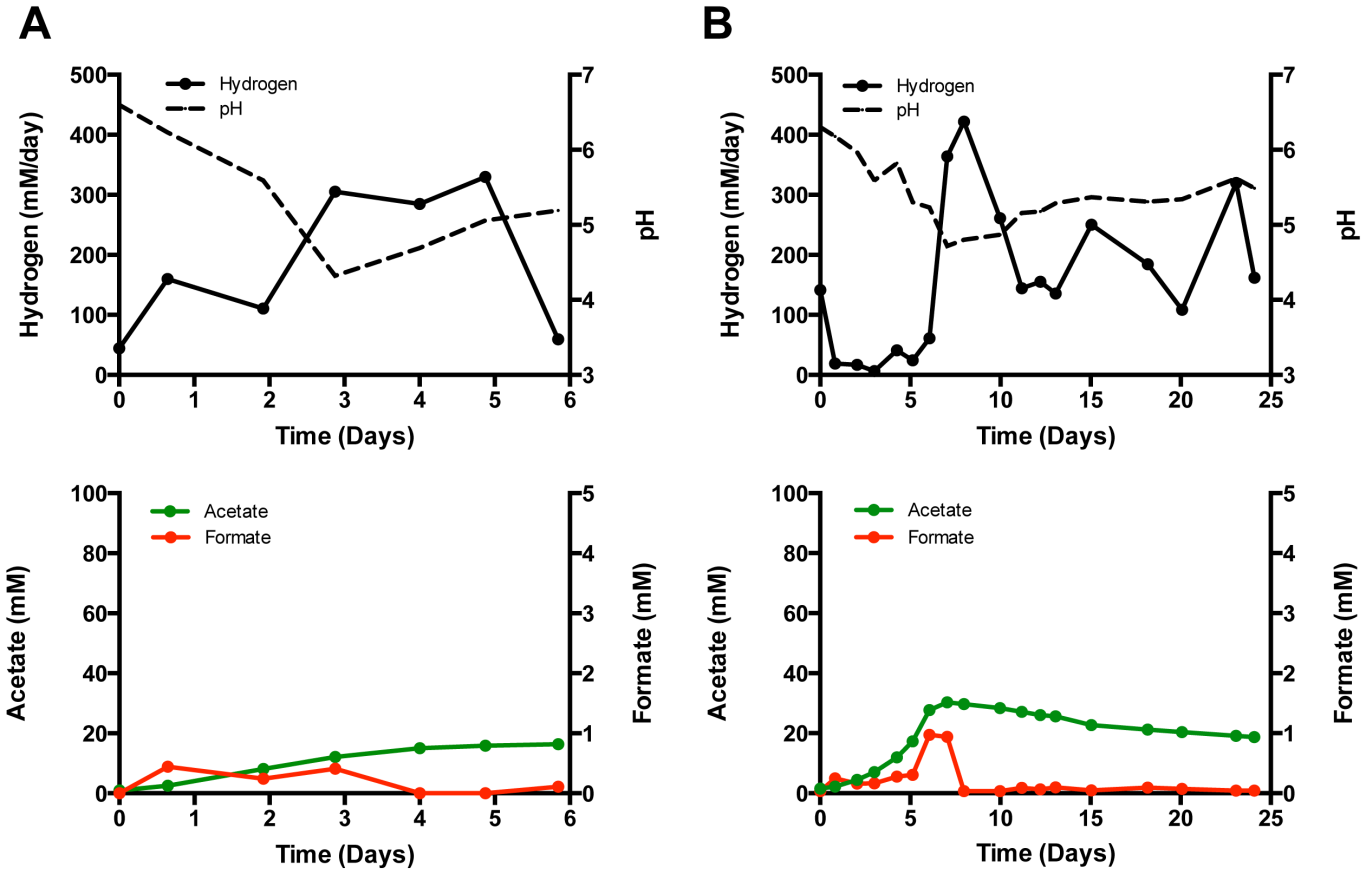
Increased hydrogen rates at acidic pH. Production of hydrogen, acetate, and formate by the electrosynthetic microbiome as pH decreases in the graphite granule biocathode of Reactor 1 poised at −600 mV vs. SHE in a bicarbonate buffer with NaBES (A), and subsequent media replacement with NaCl (B).

An acetogenic/methanogenic microbiome [2] was used to inoculate the cathode of three reactors (#1–3) with cathodes poised at −600 mV vs. SHE. Data from Reactor 1 are presented in Fig. 1 (data for the replicates are in Fig. S2). Graphite granules (25 g) from an existing biocathode were transferred to each of the new reactors supplied with bicarbonate buffered catholyte (∼pH 6.4) plus 50 mM sodium bromoethanesulfonate (NaBES) to inhibit methanogenesis. Acetate was produced from the start and continued until the pH neared 5, which is consistent with how acetogens respond to low pH [20]. Hydrogen production among the three reactors was more variable, but increased as the pH decreased and eventually reached 343 ± 25 mM/day in each reactor. Similar hydrogen production rates (343 ± 71 mM/day) but with higher rates of acetogenesis (9.7 ± 1.5 vs. 5 ± 1.8 mM/day) were obtained when the medium was replaced using 50 mM NaCl substituted for the 50 mM NaBES. Methane was not detected over 24 days in Reactors 1 & 2 when NaCl was substituted for NaBES, but methane in Reactor 3 appeared after 11 days and averaged 7.3 mM/day over the remainder of the experiment when the pH had dropped below 5 and acetogenesis had ceased.

The combination of low pH and high partial pressure of hydrogen *sans* NaBES can, in certain cases, mitigate the competitive advantage of the favorable thermodynamics and H_2_ scavenging ability of methanogens due to the commensurate kinetics of H_2_ utilization between some acetogens and methanogens [21]–[23]. With the caveat that it is strain specific, certain *Acetobacterium* spp. have been reported to outcompete certain methanogens when H_2_ is not limiting [24]. Additionally, methane production has remained non-existent or only as a minor product in microbiomes that produce carboxylic acids at pH near 5.5 [25], a pH achieved in this study. Other successful methods to minimize methanogenesis vs. acetogenesis include incubation at psychrotolerant temperatures [24] or brief treatment at higher temperatures before returning to the process temperature of the desired product [25]. The reduction in methanogenesis (none detected in several tests here) in the experiments performed without NaBES described above and some below indicate that it may be possible to specifically select for hydrogen and acetate production in the bioelectrochemical reactor. However, NaBES was used in many, but not all, experiments from hereon as a precaution against methanogenesis. Continual addition of NaBES will not be practical for the industrial implementation of microbial electrosynthesis, but the alternative methods discussed above certainly warrant further investigation to control methanogenesis [14], [21], [25]. In addition, a more conductive potassium phosphate buffered medium sparged with CO_2_ was used to lower the electrolyte resistance and maintain the cathode potential at a lower overall applied voltage, and avoid the bicarbonate buffer that is more supportive of methanogenesis. The first test with this medium was done with Reactors 4–6 (Fig. S3). Reactor 4 was inoculated from the same source used for Reactors 1–3 and Reactors 5 & 6 were inoculated from Reactor 4. In general, the response to pH and production of hydrogen and acetate was similar in this phosphate-buffered medium. Hydrogen production reached 283 ± 87.7 mM/day. Acetate in the inoculum was transferred to Reactor 4 and the rate of acetogenesis reached only ∼ 5 mM/day, but the rate was higher in Reactors 5 and 6 (12.6 and 15.3 mM/day). Formate was detected in both buffering systems, but its accumulation was varied and it is assumed to be a transient intermediate of acetate production. Sterile abiotic controls in the phosphate buffered medium produced only 0.08 and 0.49 mM/day hydrogen at pH 6.5 and 4.5, respectively, over a 4-day test in sealed reactors (Fig S4). Hydrogen was not detected when the abiotic test was repeated with the catholyte sparged with 100% CO_2_ at 15 ml/min.

**Figure 2:**
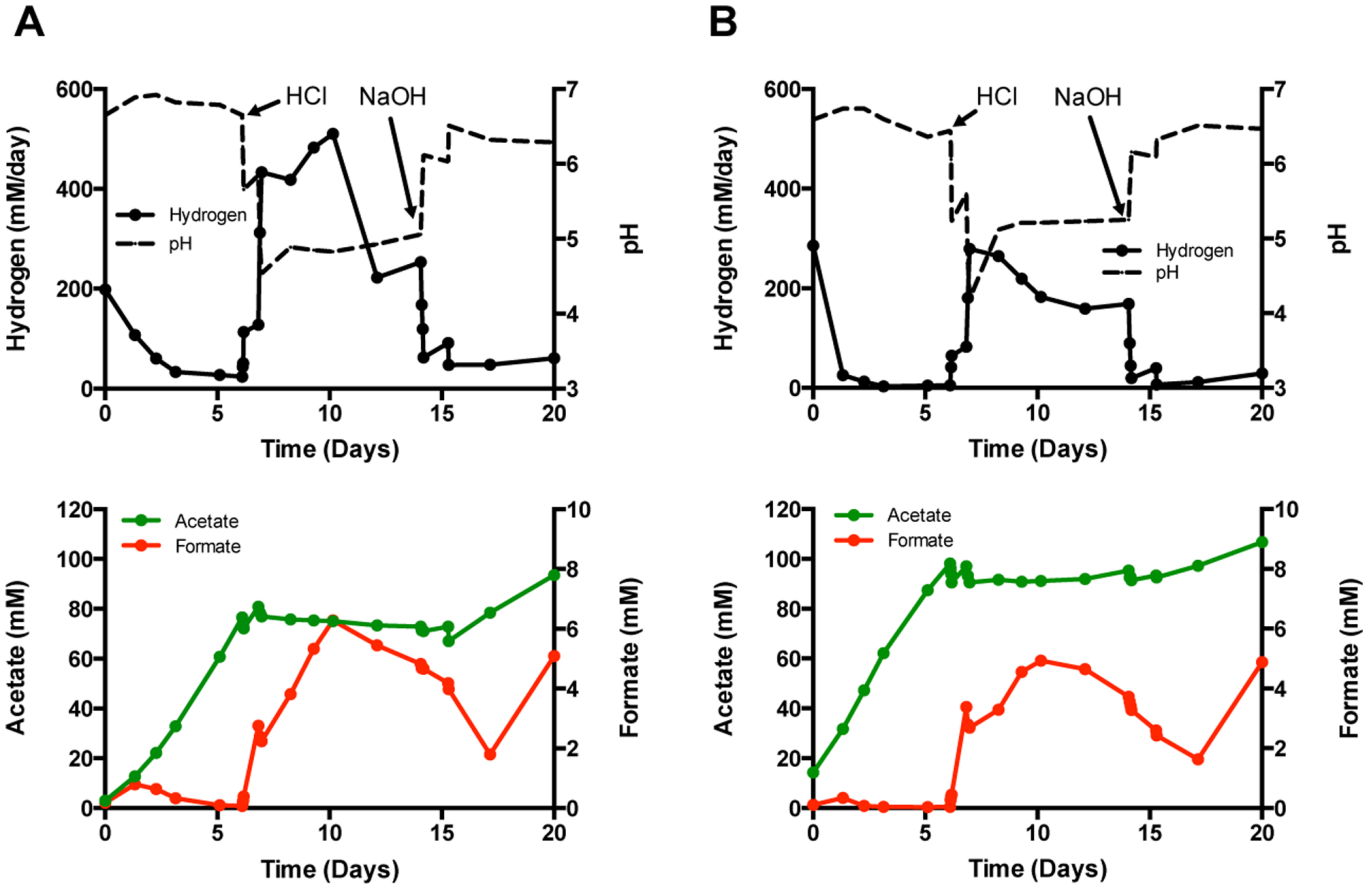
Effect of titration on production. Response of the electrosynthetic microbiome to the addition of HCl to reduce the pH, followed by titration with NaOH to re-establish near neutral pH. Reactors 4 and 5 were poised at −600 mV vs. SHE in a phosphate buffered medium with NaBES present in catholyte and anolyte.

The experiments described above were done with a salt gradient between the anode and cathode chambers of the electrochemical reactor (50 mM NaBES or 50 mM NaCl added to the cathode). This likely contributed to the pH change since without the gradient>100 mM acetic acid was needed to lower the pH significantly compared to the lowering of pH by only 30 mM acetate with the gradient. In order to determine if the response of the microbiome was due to the change in pH only, two reactors with active acetogenic microbiomes (Reactors 4 and 5) received fresh media and 50 mM NaBES added to the anode and cathode chambers. (Experiments from this point forward were always done in a phosphate buffered medium with the sodium ion concentration equal across the electrochemical cell.) Acetate was produced early in the experiment (13–19 mM/day) while hydrogen production settled below 25 mM/day and the pH stayed between 6.5 and 7 (Fig. 2). Each cathode then received titrations of HCl to lower the pH to <5 at which point acetate production ceased and hydrogen production rose to 300–500 mM per day. Formate concentration in the cathode also increased from nearly non-detectable levels to over 5 mM. The titration of NaOH raised the pH, partially restored acetate production, and slowed the production of hydrogen to less than 50 mM/day. These results are consistent with the previous experiments in that a decrease in pH is the primary cause of increased electrohydrogenesis rates in these microbiomes. This is also consistent with previous studies examining the impact of acidic pH on acetogenesis and fermentative hydrogen production [20], [26], [27].

### Yield and rate test with graphite rod electrodes

Three graphite rods (10 cm^2^ surface area) were incubated in the graphite-granule bed cathode of Reactor 4 for 48 days and exposed to 5 medium exchanges and cycles of acidic pH (<5). The rods were then transferred to serve as a defined surface area biocathode without graphite granules in three independent reactors (Reactors 7–9) with 50 ml of phosphate buffered medium plus 50 mM NaCl substituted for NaBES in each chamber.

**Figure 3:**
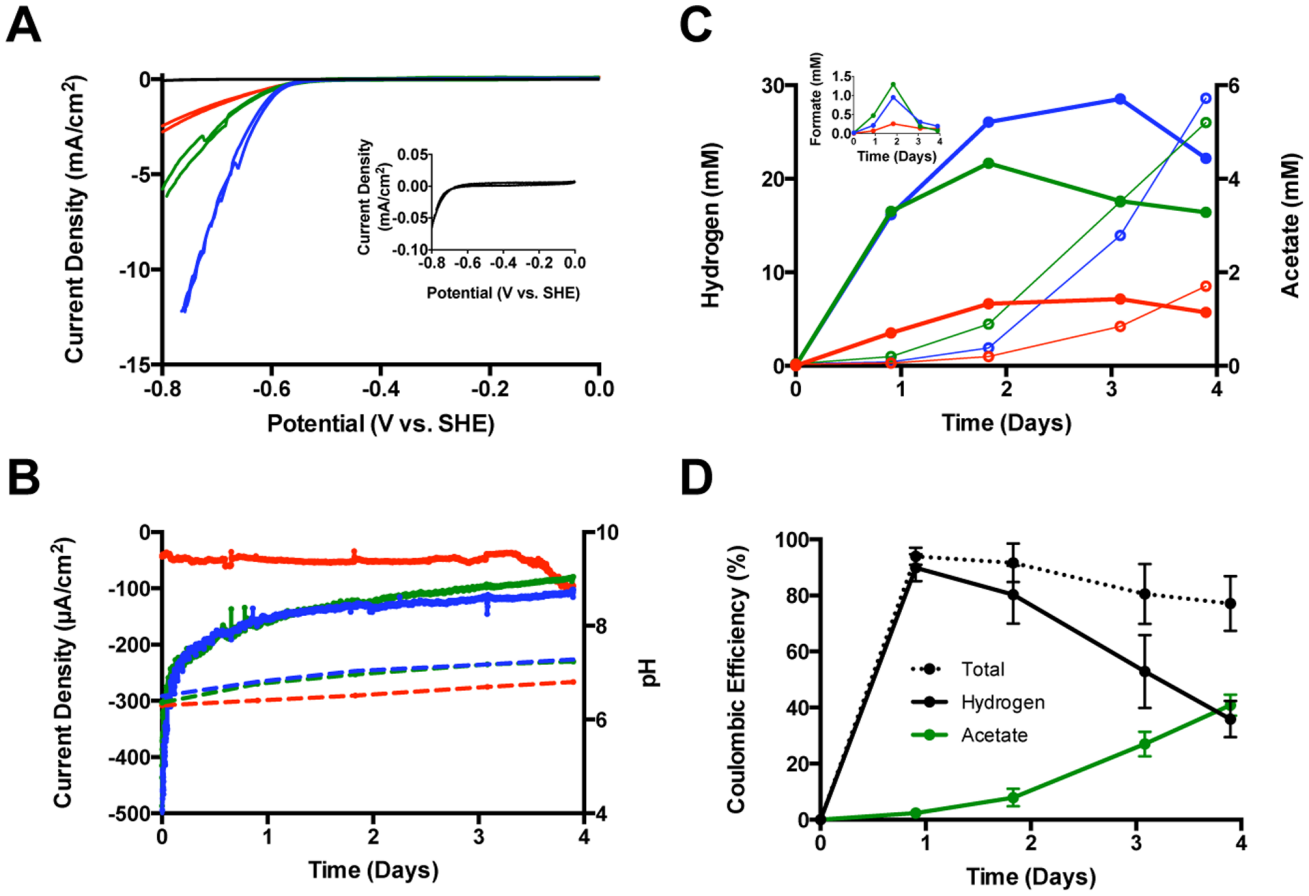
Yield and rate test. 3 graphite rod biocathodes in phosphate buffered media with NaCl. A) Cyclic voltammetry at 1 mV/sec under constant CO_2_ sparging of Reactor 7 (red), Reactor 8 (green), Reactor 9 (blue) abiotic (black). Inset: magnification of the abiotic control. Yield tests data at −600 mV vs. SHE for B–D collected with sealed reactors (no sparging, 100% CO_2_ in headspace): B) current density (solid) and pH (dashed), C) hydrogen (thick solid), acetate (thin open), and formate (solid inset), D) Coulombic efficiencies for acetate (green), hydrogen (black) and total (black dotted) with standard deviation bars.

A CV analysis was performed on all of the biocathodes after their transfer to the new reactors while the catholyte was sparged with 100% CO_2_ and the pH set near neutrality (Fig. 3A). All three biocathodes exhibited a discernible current with a potential below −500 mV vs. SHE, but they diverged as the potential was lowered further. The sweep was extended to −800 mV where the current density for two of the biocathodes reached 2.6 and 6.0 mA/cm^2^. The current density of the third reached 12.2 mA/cm^2^ at −765 mV and could not accept a lower potential due to the total applied voltage limit of the potentiostat. The differences in maximal current density may have been due to unequal microbial diversity/colonization of the three electrodes, but the overpotential for hydrogen formation on abiotic graphite electrodes was reduced by at least 250 mV with each biocathode. A sterile control with a graphite rod did not exhibit an appreciable cathodic current until the potential was below −600 mV, with a maximum current of 0.065 mA/cm^2^ at −800 mV (Fig. 3A inset). Moreover, when an active rod biocathode was exposed to oxygen (sterile air at 40 mL/min) for 20 hours, the current density decreased from 1.15 to 0.19 mA/cm^2^ at −800 mV (Fig S5). Autoclaving of the air-inactivated rod decreased the current density slightly more to 0.15 m mA/cm^2^ at −800 mV. Intriguingly, this small amount of remaining current (but no organic acid production) suggests that the dead microbiome might have left a redox-active species adsorbed to the cathode. Further examination of the mechanisms of how the electron transfer occurs within the biocathode is beyond the scope of this study, but the results indicate that a living and intact microbiome is necessary for the development of high current and organic acid production by these biocathodes.

Immediately following the CV analysis, the biocathodes were placed under 1 atm of 100% CO_2_, sealed, and poised at −600 mV vs. SHE. Productivity for two of the three biocathodes (Reactors 8 & 9) was similar over a 4-day test, but the cathode that generated the least current in the CV analysis (Reactor 7) performed poorly in relation to the others (Figs. 3B & 3C). Although acetate production was detected during day 1 of the yield test, the accumulation of hydrogen was much higher (Fig. 3C). A small amount of formate peaked with each biocathode on day 2 (Fig. 3C inset). Maximal production rates normalized to the surface area of the electrodes are presented in Table 1. A strong cathodic current discharge was exhibited by the more robust biocathodes at time zero, and each of these then generated a current of>100 µA/cm^2^ over the next 4 days (Fig. 3C). Reactor 7 did not show such an initial discharge and the current remained near 40 µA/cm^2^ until after day 3 when it began to accelerate, perhaps indicating the onset of growth on the electrode. By day 4, the three biocathodes were generating 92.0 ± 11.8 µA/cm^2^ and the experiment was terminated to measure biomass (protein) on the electrodes. Protein densities were comparable at this time (14.3 ± 0.9 µg/cm^2^) and the average current density to protein for the three biocathodes was of 6.5 ± 1.2 A/g. The coulombic efficiency for each reactor was similar with nearly 90% to hydrogen early on and over 40% to acetate at the end of the experiment (Fig. 3D). Overall coulombic efficiency was maximal at day 1 (94% ± 3%) and somewhat decreased by day 4 (77% ± 9.8%), with perhaps the remainder of the electrons captured as biomass. No methane was detected in any of the sealed reactors despite the lack of NaBES in the medium.

**Table 1:** Maximum productivity of graphite rod biocathodes poised at −600 mV vs. SHE normalized to surface area.

Biocathode	H_2_	Formate	Acetate
	g/m^2^/day	g/m^2^/day	g/m^2^/day
**Reactor 7**	0.39	0.46	3.16
**Reactor 8**	1.82	2.03	6.28
**Reactor 9**	1.78	1.82	10.84

### Further increases in hydrogen and acetate production

**Figure 4:**
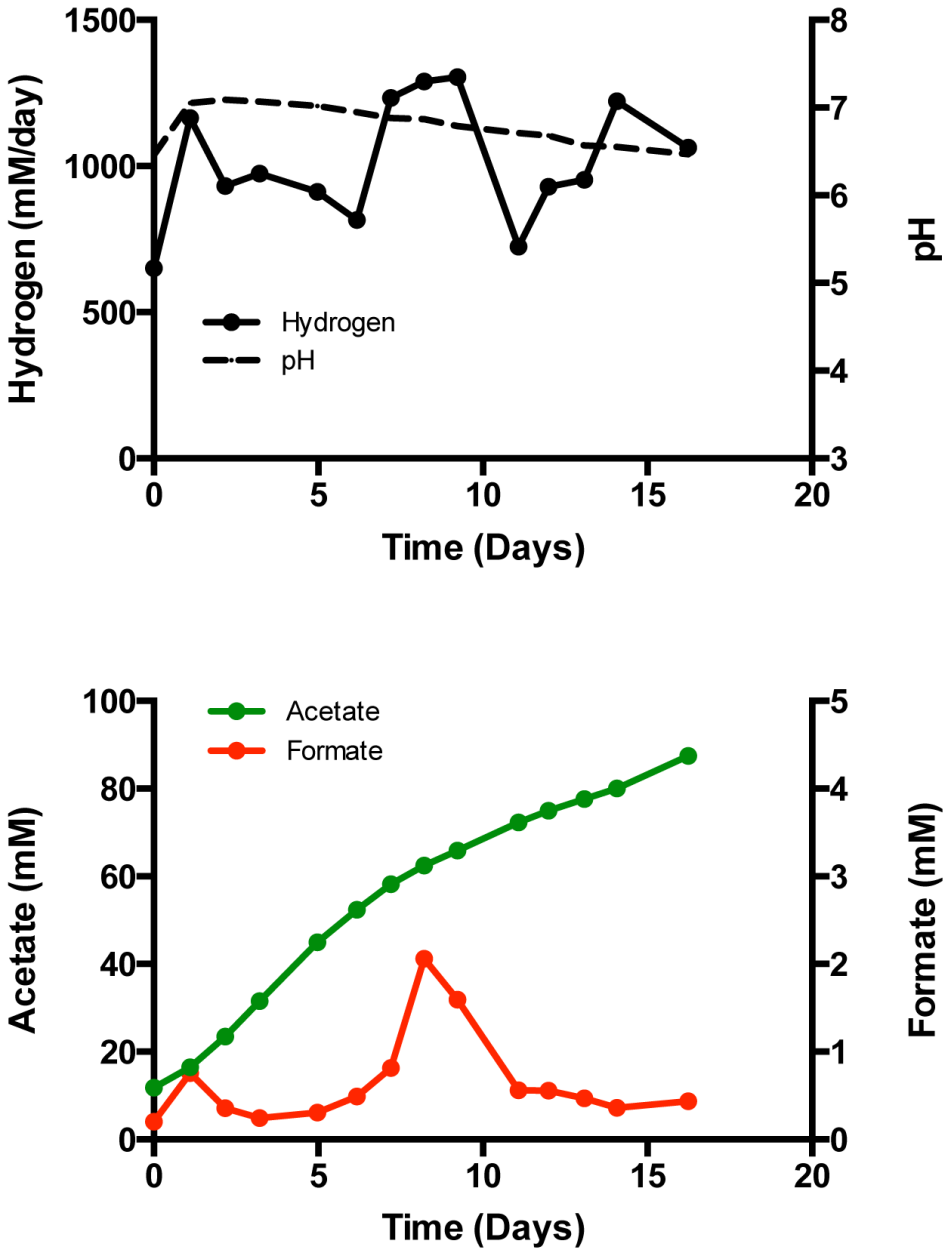
Sustained hydrogen production at higher pH after enrichment. Reactor 4 at −600 mV vs. SHE in phosphate buffered media with NaBES in the anolyte and catholyte.

Following multiple replacements of the catholyte and repeated exposure of the microbiome to acidic pH, it became apparent that hydrogen production would remain relatively high even at near neutral pH. This was first observed with Reactor 4 after 6 medium exchanges and low pH cycles with the cathode always poised at −600 mV vs. SHE (Fig. 4). Hydrogen was produced at>1000 mM/day even though the pH never dropped below 6.5 in that experiment. Subsequent transfers of the microbiome continued to exhibit high hydrogen production. This suggested that the microbial population on the granules had adapted/grown to produce more hydrogen regardless of the operating pH as long as at one point in the microbiome's development the community was exposed to acidic pH. At this time, the electrode granule and planktonic communities of Reactor 4 were sampled for phylogenetic analysis (see below).

**Figure 5:**
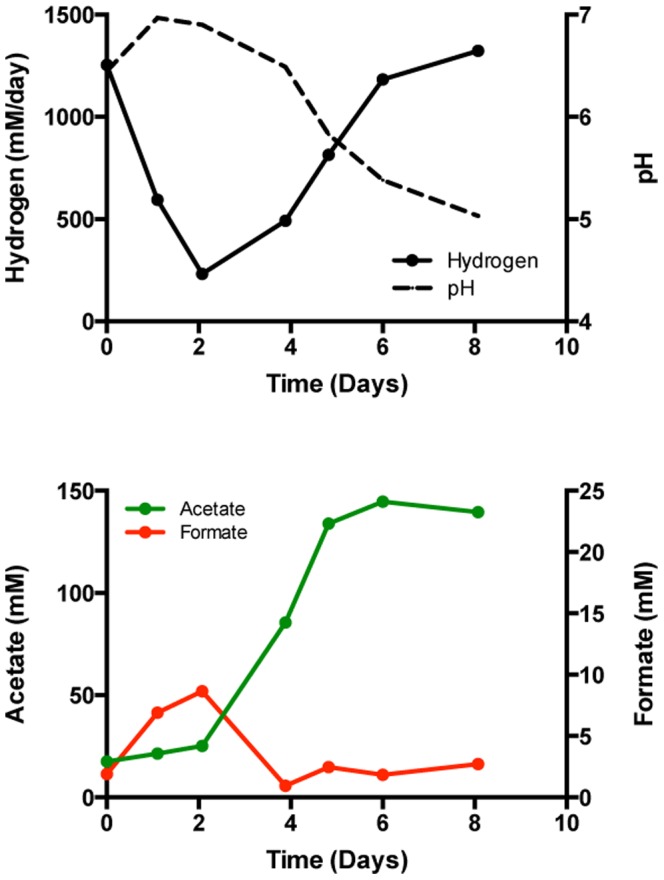
Increased rates at lower potential. Increased acetate and hydrogen production of the microbiome in the larger Reactor 10 poised at −800 mV vs. SHE in phosphate buffered media with NaBES in the anolyte and catholyte.

With these comparatively high levels of productivity and current density, it was not possible to examine the biocathode when poised at a lower potential due to the total applied voltage limitation of the potentiostat. To address this, a different type of reactor (Reactor 10) with a larger membrane and working volume (100 mL) was inoculated with granules from Reactor 4. Relieving the membrane limitation allowed the reactor to be operated at −800 mV vs. SHE. Under these conditions, high hydrogen and organic acid production was achieved as the pH decreased (Fig. 5). The hydrogen production rate was ∼1250 mM/day and formate production was 4.5 mM/day immediately at the start of this experiment; both results were to be expected with an inoculum of populated granules from a reactor that was producing high amounts of hydrogen and acetate. Over the first two days, the detected hydrogen production rate decreased while acetate accumulation increased only modestly. The pH then began to decrease sharply as both hydrogen and acetate production increased concomitantly. Acetate production reached a maximum rate of 51.6 mM/day and ceased when the pH dropped below ∼5.5. At the lower pH, the hydrogen production rate exceeded 1300 mM/day. Following two more cycles of medium replacement, high hydrogen rates persisted along with 16.9 mM/day formate and 28.7 mM/day acetate. The potential of the cathode was modulated to avoid exceeding the current limit of the potentiostat. Acetate and hydrogen production remained high with the cathode poised at −750 mV (>1000 mM/day hydrogen and a maximum rate of 51.9 mM/day acetate)(Fig. S6). A table summarizing the conditions and maximum productivities of all reactors is available in the Table S1.

*Acetobacterium woodii* has been shown to produce a maximum of 123 mM/day acetate when incubated under a partial hydrogen pressure of 1700 mbar [27]. This was the fastest rate of autotrophic acetogenesis reported until the same group demonstrated that *A. woodii* would produce 421 mM/day in a nutrient enriched medium stirred at 1200 rpm under 40% hydrogen [28]. The authors also genetically transformed *A. woodii* to overexpress phosphotransacetylase and acetate kinase or the four THF dependent enzymes of the Wood-Ljungdahl pathway in order to overcome carbon flux bottlenecks. These mutants produced acetate at 480 mM/day in the same medium and conditions. The current densities and product formation by the electrosynthetic microbiota from the current study indicate that electron flux from an electrode to a microbial community is similar to these rates of hydrogenotrophic acetogenesis, albeit without the energy required to pressurize or stir the reactor. For example, the electrosynthetic microbiome has produced>50 mM/day acetate, while an additional 325 mM/day could potentially be generated based on the electron equivalents for maximum hydrogen production rates observed using the system.

### Potential roles of members of the microbiome

**Figure 6:**
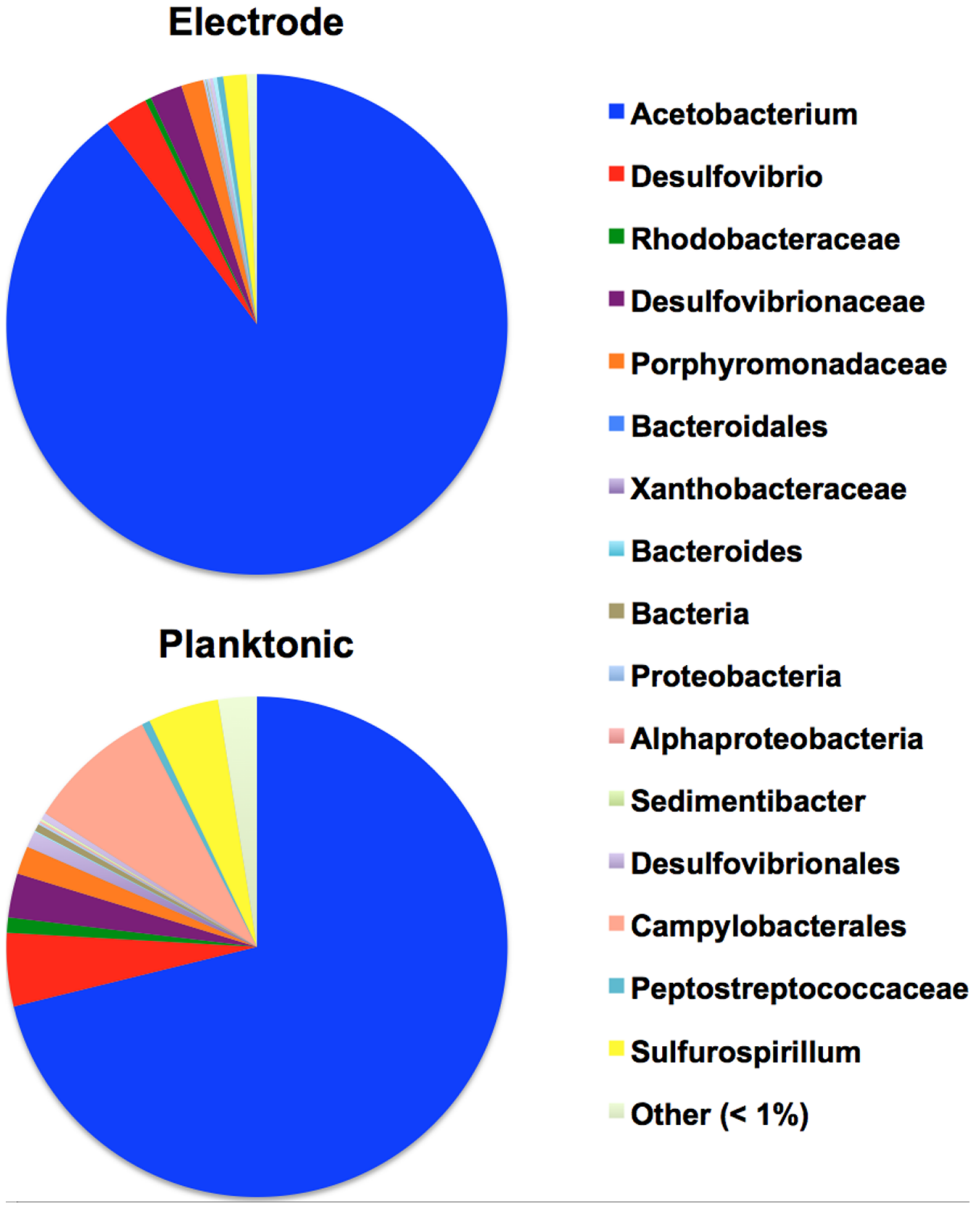
Microbiome composition. Phylogenetic assessment of the electrode-attached cells and planktonic cells of the electrosynthetic microbiome after enrichment following repeated exposure to acidic pH.

**Figure 7:**
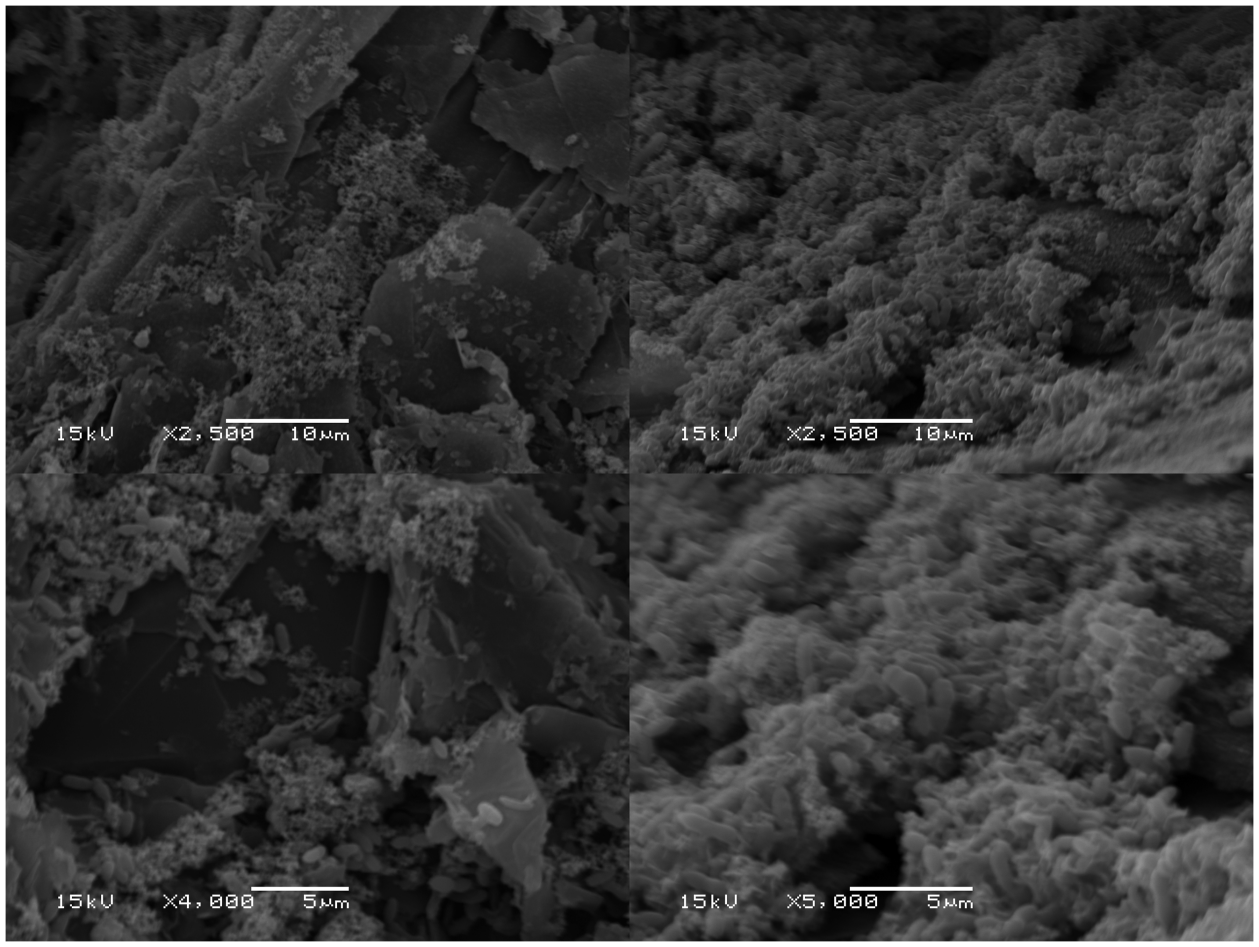
Biofilm establishment. SEM images of a 1 month old (left) and a 3-month-old (right) biofilm on graphite granules in phosphate buffered media poised at −600 mV vs. SHE. Scale bars are 10 µm (top) and 5 µm (bottom).

Microbial communities growing on the surface of the graphite granule electrode and the planktonic community within the cathode chamber were characterized using 16S rRNA amplicon sequencing on the samples taken from Reactor 4 that were used to inoculate Reactor 10. Only 5 abundant taxa were observed, with the electrode and planktonic communities being dominated by an *Acetobacterium* sp., particularly on the electrode, where it comprised 90% of the relative abundance (Fig. 6). The composition is similar to what was observed for this microbiome in earlier studies [1], [2] but with a higher proportion of *Acetobacterium* obtained in this study. The remaining members of the community on the electrode included two *Desulfovibrio* spp., a *Sulfurospirillum* sp. and a member of the Porphyromonadaceae. SEM analysis of the microbiome on the cathode near the time of the samples that were acquired for 16S rRNA characterization revealed a microbial biofilm on the electrode surface that included morphologies consistent with those expected for the genera detected by sequence analysis (Fig. 7).

The paradigm to date with pure cultures of electrosynthetic acetogens (e.g. *Sporomusa ovata* or *Clostridium ljungdahlii*) is that these organisms capture electrons directly from an electrode and synthesize acetate [3], [4]. Direct electron transfer (DET) to *Acetobacterium* could possibly be contributing to the productivity of the microbiome, but it is difficult to discern DET to acetogenesis when hydrogen is concomitantly produced in such high amounts. Current densities are sufficiently high at cathodic potentials so that CV scans of the biocathodes are dominated by the hydrogen reaction, and hydrogen forming at the electrode surface is readily visualized bubbling vigorously off of the biocathode surface when the pH is low or after the community has grown/adapted to higher hydrogen production following an acidic pH challenge (Fig. S7 and Movie S1). So while DET to the acetogens in the microbiome has not yet been identified under the conditions tested, DET coupled to microbially driven hydrogen may occur, as previously shown with *Desulfovibrio* sp. on carbon electrodes [29].

The development of elevated hydrogen production and increasing acetogenesis with *Acetobacterium* dominating the cathode strongly implicates the acetogen alone as the electrosynthetic producer of hydrogen and organic acids. Hydrogenases from active and inactive cells may contribute and could be responsible for the cathodic signal and reduced hydrogen overpotential observed by CV. On the electrode the acetogen may generate hydrogen that is then converted to acetate following intraspecies transfer. However, the genus has not yet been shown to perform DET in pure culture or generate hydrogen gas on a cathode. More work is needed to determine how this Gram-positive microorganism contributes to the output of the electrosynthetic microbiome in addition to acetogenesis. Alternatively, the microbiome may rely on interspecies interactions in addition to DET. *Desulfovibrio* spp. have been found in a hydrogen producing biocathode [30] and have been shown to directly oxidize a cathode and produce hydrogen [29], [31]. This may require the capture of electrons by outer membrane cytochromes (OMCs) analogous to those found in Gram-negative microorganisms capable of reducing anodes, such as *Geobacter* and *Shewanella* [32]–[35]. *Desulfovibrio* spp. possess OMCs plus soluble cytochromes and hydrogenases in the periplasm [36]–[38] that may readily facilitate the electron transport from electrode to hydrogenase. *Desulfovibrio* spp. are not known to grow autotrophically and often require an external source of acetate, but this could be supplied by *Acetobacterium*. In fact, the addition of acetate has been shown to enhance the startup of a hydrogen producing mixed-community biocathode [39] and *Acetobacterium* was reported to supply acetate to *Desulfovibrio* in co-cultures and enrichments growing with H_2_, CO_2_, and sulfate [40]. Other members of the microbiome may perform the roles proposed for *Desulfovibrio* and *Acetobacterium* and more study is needed, but the known physiology of these genera renders this a plausible hypothesis.

Formate metabolism and interspecies (or intraspecies) transfer may also play an important role in the metabolism of the electrosynthetic microbiome. For example, *Acetobacterium* will utilize formate and *Sulfurospirillum* has been shown to utilize hydrogen or formate when acetate is present as a carbon source [41]. Recently a CO_2_ reductase in *Acetobacterium woodii* was shown to catalyze the reversible and direct interconversion of hydrogen and carbon dioxide to formate, independent of other energy carriers [42]. Additionally, under sulfate-limiting conditions, it is thermodynamically favorable for sulfate-reducing bacteria, some of which also possess a CO_2_ reductase [42], to produce formate from hydrogen and CO_2_ provided there are acetogens or methanogens that can consume it fast enough [43]. While DET to formate has not yet been demonstrated in whole cells, protein film voltammetry techniques have shown formate dehydrogenase to be capable of DET to and from carbon electrodes at rapid rates [44]. *Desulfovibrio* spp. also contain soluble formate dehydrogenases in the periplasm [38], [43], [45], to which OMCs could transfer electrons as described above. Nevertheless, the transient nature of formate accumulation and the known physiology of the organisms detected implicate formate as a possible route for interspecies metabolite transfer by the electrosynthetic microbiome.

## Conclusion

Repeated exposure to acidic pH resulted in up to 2.6 kg/m^3^/day hydrogen (1 kg H_2_ ≈ 1 GGE) production by an electrosynthetic microbiome. High acetate production occurred (up to 3.1 kg/m^3^/day) when the pH was>5, with 1.5 kg of CO_2_ captured per kg acetate produced. CV analysis indicated a lowering of the overpotential at the cathode, and current densities as high as 12.2 mA/cm^2^. Oxygen and autoclave treatment ameliorated the cathodic current. *Acetobacterium* may solely facilitate these reactions but alternatively, interspecies interactions by the microbiome may be required to produce high levels of hydrogen and organic acids during microbial electrosynthesis.

## Supporting Information

Figure S1 **Inoculation scheme of the 10 reactors used in this study.** Reactors 1-4 were inoculated from a previous electrosynthetic microbiome. NaBES or NaCl at 50 mM was added to the cathode (C) or anode and cathode (A + C) of each reactor. Reactors 1–3 were operated with bicarbonate buffered medium with 50 mM NaBES in the catholyte. They were replenished with fresh media wherein 50 mM NaCl was substituted for the NaBES in the catholyte. Reactor 4 was operated with a phosphate buffered medium with 50 mM NaBES in the catholyte or in both catholyte and anolyte. Reactors 5 and 6 were two replicate reactors inoculated from Reactor 4 and under the same conditions. Yield tests and CV were performed in Reactors 7–9 which contained phosphate buffered media with 50 mM NaCl in the anolyte and catholyte and rods initially incubated in Reactor 4. Reactor 10 was the larger membrane reactor inoculated with granules from Reactor 4 and poised at lower potentials in phosphate buffered media with 50 mM NaBES in the anolyte and catholyte.
(PDF)

Figure S2 **Replicates of Reactor 1 and the conditions presented in** **Figure 1** **.** Biocathodes of Reactors 2 (A and B) and 3 (C and D) were incubated in bicarbonate buffered media with 50 mM NaBES (A and C) or 50 mM NaCl (B and D) in the catholyte and poised at −600 mV vs. SHE.
(PDF)

Figure S3 **Transferability and replication of the electrosynthetic microbiome.** Granules were transferred from Reactor 4 (A) into Reactors 5 (B) and 6 (C) and exposed to lowered pH in phosphate buffered media containing 50 mM NaBES in the catholyte −600 mV vs. SHE.
(PDF)

Figure S4 **Abiotic controls.** Hydrogen production (solid lines) in low and high pH (dashed) sterile and sealed reactors. Graphite granule cathodes were poised at −600 mV vs. SHE in phosphate-buffered medium with 50 mM sodium BES and with (blue) or without (red) 100 mM acetic acid.
(PDF)

Figure S5 **Inactivation of an active biocathode.** Cyclic voltammogram of an active rod biocathode in phosphate buffered medium at pH = 6.3 with 50 mM NaCl in the anolyte and catholyte, and 100% CO_2_ sparge (black). The active rod was exposed to sterile flowing air (40 mL/min) for 20 hours and the scan was repeated under 100% CO_2_ sparge (blue). The O_2_ inactivated rod was then autoclaved on a gravity cycle for 30 min and the scan was repeated again under 100% CO_2_ sparge (red). An abiotic sterile control (gray) and the autoclave and O_2_ inactivation treatments showed far less cathodic current densities than the active biocathode.
(PDF)

Figure S6 **Improved production at lower potential.** Sequential media replacements (A and B) of phosphate buffer medium with 50 mM BES in the anolyte and catholyte in Reactor 10 poised at −800 mV vs. SHE unless otherwise indicated.
(PDF)

Figure S7 **Screen shot of hydrogen gas evolving off of biocathode.** Video screenshot of a biocathode in phosphate buffered media poised at −600 mV vs. SHE.
(PDF)

Table S1 **Summarized parameters and maximum productivities for the reactors in this study.** Maximum productivities are in mM/day, and g/m^2^/day is in parenthesis for the rods. Presence of NaBES or NaCl in the catholyte or catholyte and anolyte is noted by C or C & A, respectively.
(PDF)

Movie S1 **Brief video of the biocathode evolving hydrogen gas at −600 mV vs. SHE.**
(MOV)
