# Supplementary material for: Influence of Acidic pH on Hydrogen and Acetate Production by an Electrosynthetic Microbiome
Source: PLoS One. 2014 Oct 15;9(10):e109935. doi: 10.1371/journal.pone.0109935 (PMC4198145; doi:10.1371/journal.pone.0109935)
Supplement: Figure S1 — Inoculation scheme of the 10 reactors used in this study. Reactors 1-4 were inoculated from a previous electrosynthetic microbiome. NaBES or NaCl at 50 mM was added to the cathode (C) or anode and cathode (A + C) of each reactor. Reactors 1–3 were operated with bicarbonate buffered medium with 50 mM NaBES in the catholyte. They were replenished with fresh media wherein 50 mM NaCl was substituted for the NaBES in the catholyte. Reactor 4 was operated with a phosphate buffered medium with 50 mM NaBES in the catholyte or in both catholyte and anolyte. Reactors 5 and 6 were two replicate reactors inoculated from Reactor 4 and under the same conditions. Yield tests and CV were performed in Reactors 7–9 which contained phosphate buffered media with 50 mM NaCl in the anolyte and catholyte and rods initially incubated in Reactor 4. Reactor 10 was the larger membrane reactor inoculated with granules from Reactor 4 and poised at lower potentials in phosphate buffered media with 50 mM NaBES in the anolyte and catholyte. (PDF) [file pone.0109935.s001.pdf]

Supplemental Data

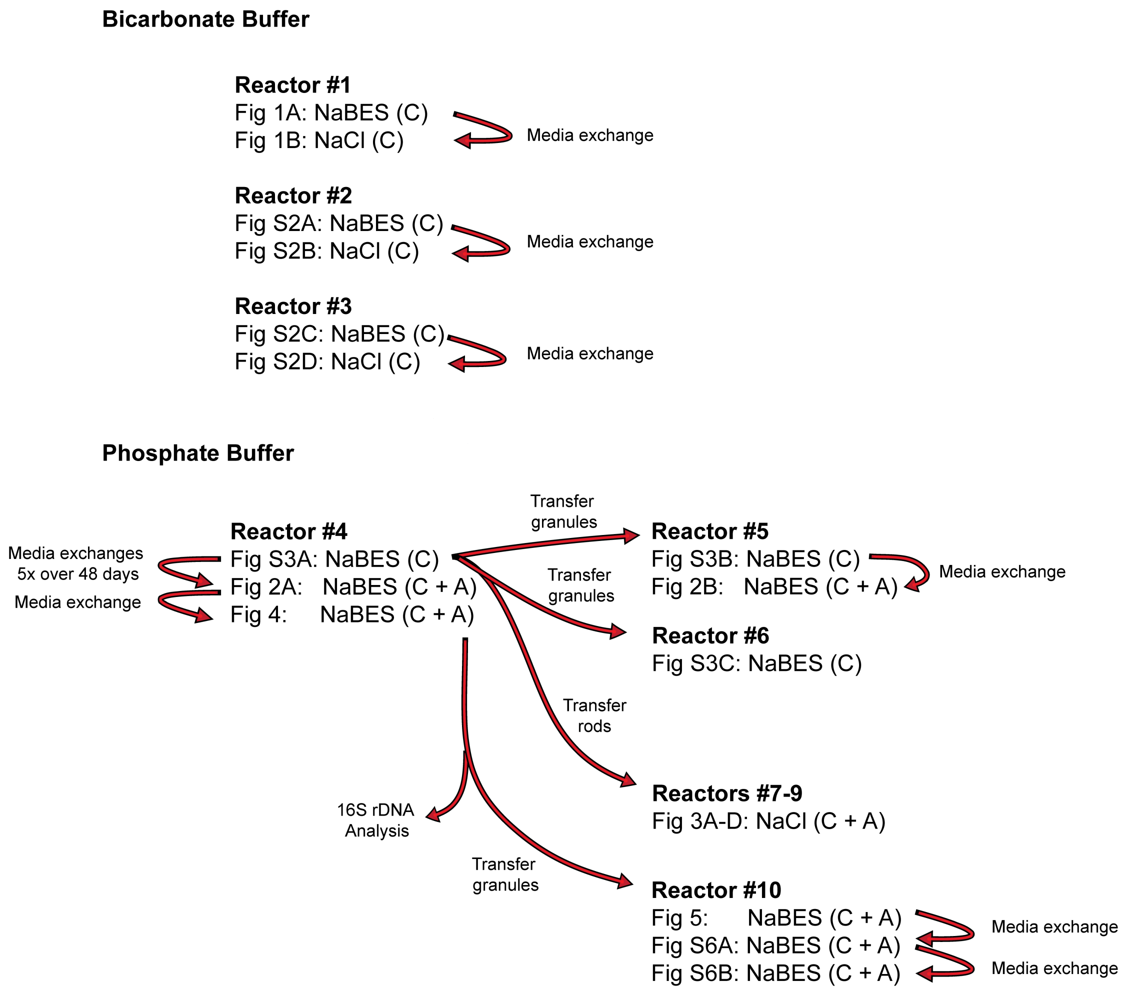

Figure S1. Inoculation scheme of the 10 reactors used in this study. Reactors 1-4 were inoculated from a previous electrosynthetic microbiome. NaBES or NaCl at 50 mM was added to the cathode (C) or anode and cathode (A + C) of each reactor. Reactors 1-3 were operated with bicarbonate buffered medium with 50 mM NaBES in the catholyte. They were replenished with fresh media wherein 50 mM NaCl was substituted for the NaBES in the catholyte. Reactor 4 was operated with a phosphate buffered medium with 50 mM NaBES in the catholyte or in both catholyte and anolyte. Reactors 5 and 6 were two replicate reactors inoculated from Reactor 4 and under the same conditions. Yield tests and CV were performed in Reactors 7-9 which contained phosphate buffered media with 50 mM NaCl in the anolyte and catholyte and rods initially incubated in Reactor 4. Reactor 10 was the larger membrane reactor inoculated with granules from Reactor 4 and poised at lower potentials in phosphate buffered media with 50 mM NaBES in the anolyte and catholyte.
