# Supplementary material for: Influence of Acidic pH on Hydrogen and Acetate Production by an Electrosynthetic Microbiome
Source: PLoS One. 2014 Oct 15;9(10):e109935. doi: 10.1371/journal.pone.0109935 (PMC4198145; doi:10.1371/journal.pone.0109935)
Supplement: Figure S2 — Replicates of Reactor 1 and the conditions presented in Figure 1 . Biocathodes of Reactors 2 (A and B) and 3 (C and D) were incubated in bicarbonate buffered media with 50 mM NaBES (A and C) or 50 mM NaCl (B and D) in the catholyte and poised at −600 mV vs. SHE. (PDF) [file pone.0109935.s002.pdf]

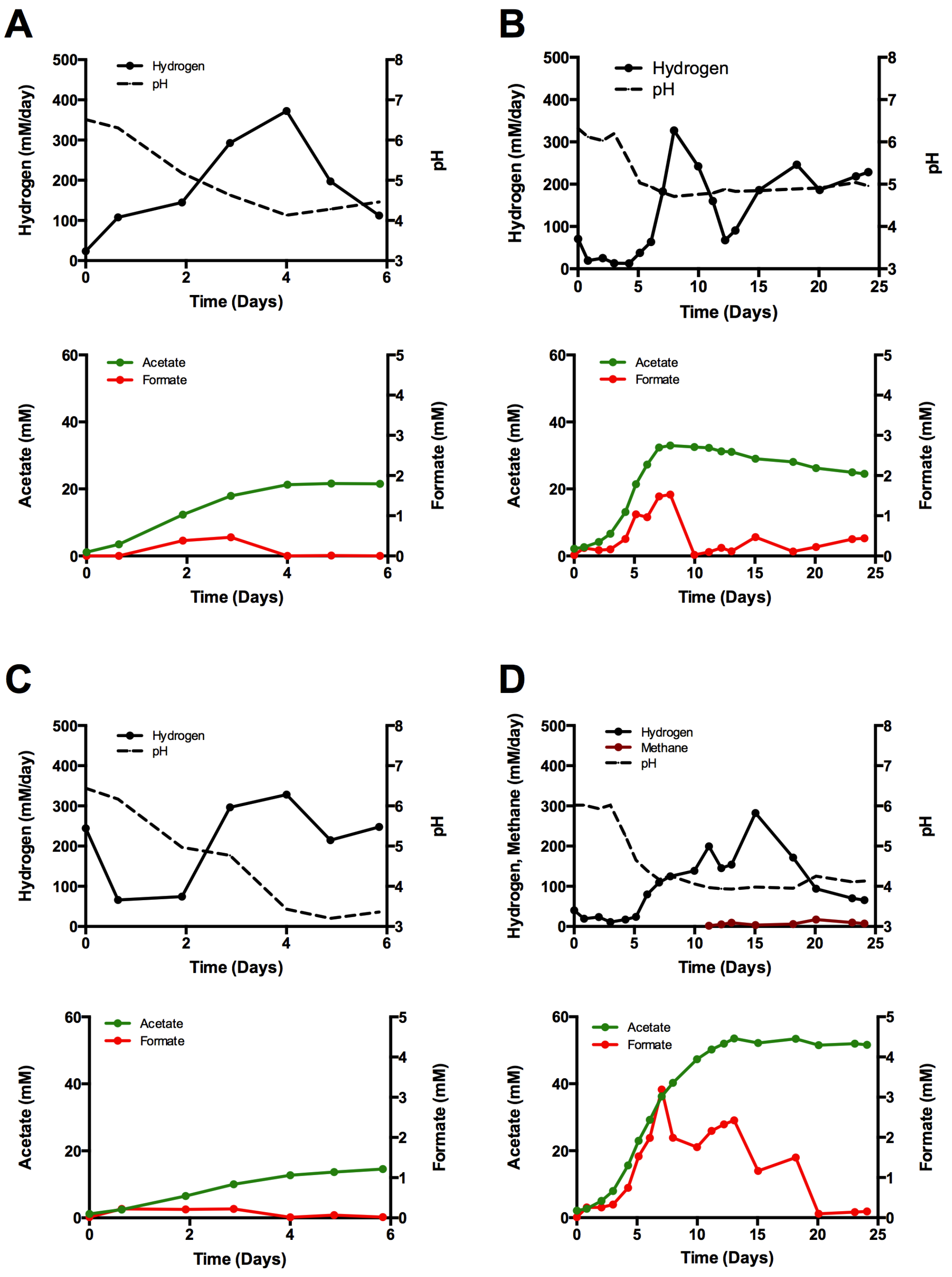

Figure S2. Replicates of Reactor 1 and the conditions presented in Figure 1. Biocathodes of Reactors 2 (A and B) and 3 (C and D) were incubated in bicarbonate buffered media with 50 mM NaBES (A and C) or 50 mM NaCl (B and D) in the catholyte and poised at -600 mV vs. SHE.
