# Supplementary material for: Influence of Acidic pH on Hydrogen and Acetate Production by an Electrosynthetic Microbiome
Source: PLoS One. 2014 Oct 15;9(10):e109935. doi: 10.1371/journal.pone.0109935 (PMC4198145; doi:10.1371/journal.pone.0109935)
Supplement: Figure S3 — Transferability and replication of the electrosynthetic microbiome. Granules were transferred from Reactor 4 (A) into Reactors 5 (B) and 6 (C) and exposed to lowered pH in phosphate buffered media containing 50 mM NaBES in the catholyte −600 mV vs. SHE. (PDF) [file pone.0109935.s003.pdf]

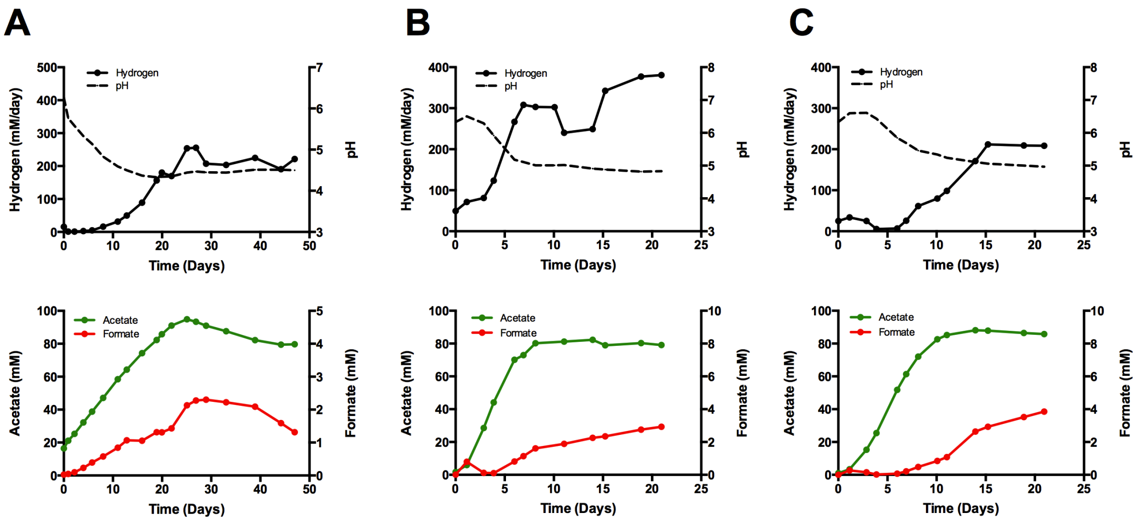

Figure S3. Transferability and replication of the electrosynthetic microbiome. Granules were transferred from Reactor 4 (A) into Reactors 5 (B) and 6 (C) and exposed to lowered pH in phosphate buffered media containing 50 mM NaBES in the catholyte -600 mV vs. SHE.
