# Supplementary material for: Influence of Acidic pH on Hydrogen and Acetate Production by an Electrosynthetic Microbiome
Source: PLoS One. 2014 Oct 15;9(10):e109935. doi: 10.1371/journal.pone.0109935 (PMC4198145; doi:10.1371/journal.pone.0109935)
Supplement: Figure S4 — Abiotic controls. Hydrogen production (solid lines) in low and high pH (dashed) sterile and sealed reactors. Graphite granule cathodes were poised at −600 mV vs. SHE in phosphate-buffered medium with 50 mM sodium BES and with (blue) or without (red) 100 mM acetic acid. (PDF) [file pone.0109935.s004.pdf]

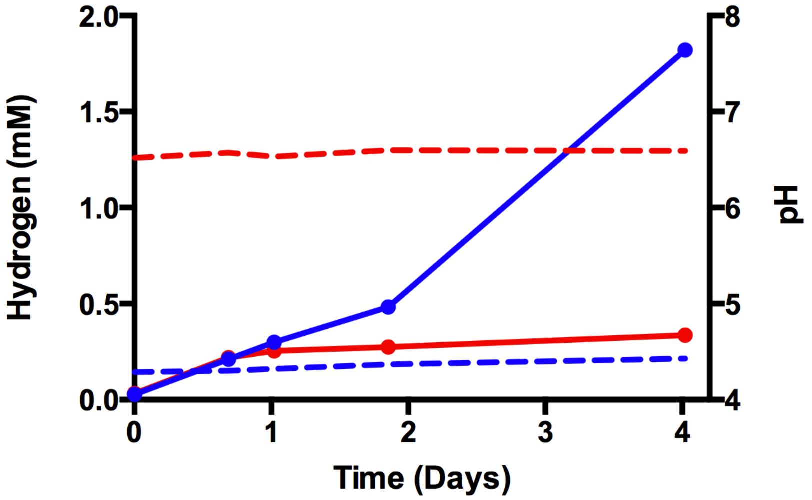

Figure S4. Abiotic controls. Hydrogen production (solid lines) in low and high pH (dashed) sterile and sealed reactors. Graphite granule cathodes were poised at -600 mV vs. SHE in phosphate-buffered medium with 50 mM sodium BES and with (blue) or without (red) 100 mM acetic acid.
