# Supplementary material for: Influence of Acidic pH on Hydrogen and Acetate Production by an Electrosynthetic Microbiome
Source: PLoS One. 2014 Oct 15;9(10):e109935. doi: 10.1371/journal.pone.0109935 (PMC4198145; doi:10.1371/journal.pone.0109935)
Supplement: Figure S5 — Inactivation of an active biocathode. Cyclic voltammogram of an active rod biocathode in phosphate buffered medium at pH = 6.3 with 50 mM NaCl in the anolyte and catholyte, and 100% CO2 sparge (black). The active rod was exposed to sterile flowing air (40 mL/min) for 20 hours and the scan was repeated under 100% CO2 sparge (blue). The O2 inactivated rod was then autoclaved on a gravity cycle for 30 min and the scan was repeated again under 100% CO2 sparge (red). An abiotic sterile control (gray) and the autoclave and O2 inactivation treatments showed far less cathodic current densities than the active biocathode. (PDF) [file pone.0109935.s005.pdf]

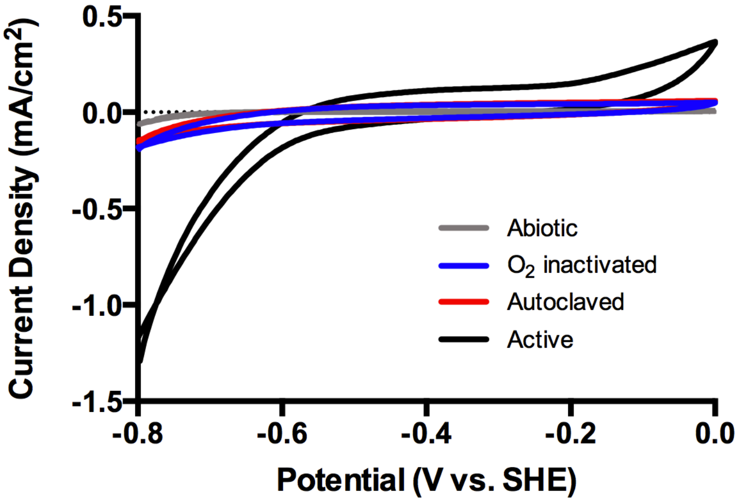

Figure S5. Inactivation of an active biocathode. Cyclic voltammogram of an active rod biocathode in phosphate buffered medium at pH=6.3 with 50 mM NaCl in the anolyte and catholyte, and 100% CO_2 sparge (black). The active rod was exposed to sterile flowing air (40 mL/min) for 20 hours and the scan was repeated under 100% CO_2 sparge (blue). The O_2 inactivated rod was then autoclaved on a gravity cycle for 30 min and the scan was repeated again under 100% CO_2 sparge (red). An abiotic sterile control (gray) and the autoclave and O_2 inactivation treatments showed far less cathodic current densities than the active biocathode.
