# Supplementary material for: Influence of Acidic pH on Hydrogen and Acetate Production by an Electrosynthetic Microbiome
Source: PLoS One. 2014 Oct 15;9(10):e109935. doi: 10.1371/journal.pone.0109935 (PMC4198145; doi:10.1371/journal.pone.0109935)
Supplement: Figure S6 — Improved production at lower potential. Sequential media replacements (A and B) of phosphate buffer medium with 50 mM BES in the anolyte and catholyte in Reactor 10 poised at −800 mV vs. SHE unless otherwise indicated. (PDF) [file pone.0109935.s006.pdf]

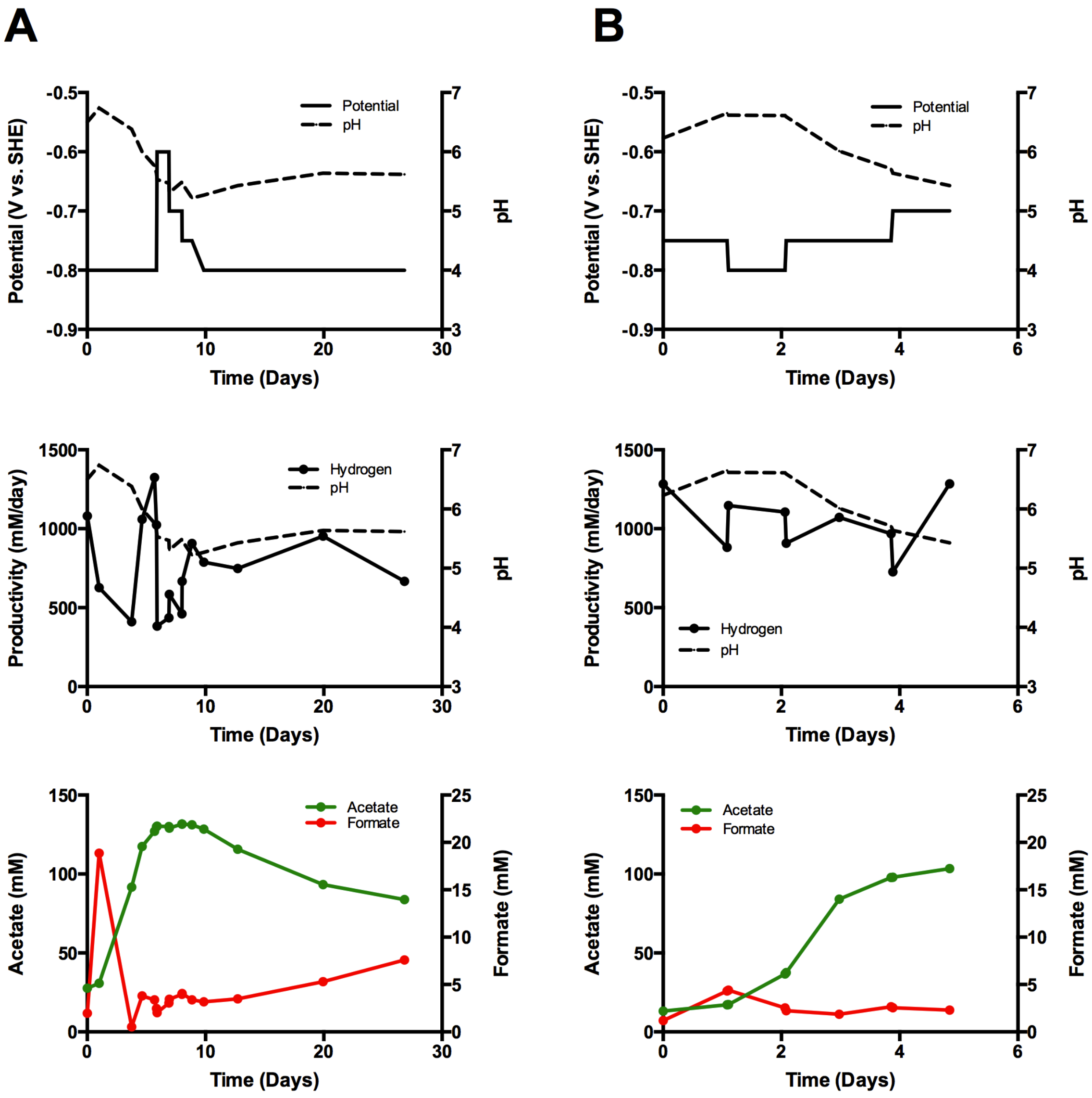

Figure S6. Improved production at lower potential. Sequential media replacements (A and B) of phosphate buffer medium with 50 mM BES in the anolyte and catholyte in Reactor 10 poised at -800 mV vs. SHE unless otherwise indicated.
