# Supplementary material for: Influence of Acidic pH on Hydrogen and Acetate Production by an Electrosynthetic Microbiome
Source: PLoS One. 2014 Oct 15;9(10):e109935. doi: 10.1371/journal.pone.0109935 (PMC4198145; doi:10.1371/journal.pone.0109935)
Supplement: Figure S7 — Screen shot of hydrogen gas evolving off of biocathode. Video screenshot of a biocathode in phosphate buffered media poised at −600 mV vs. SHE. (PDF) [file pone.0109935.s007.pdf]

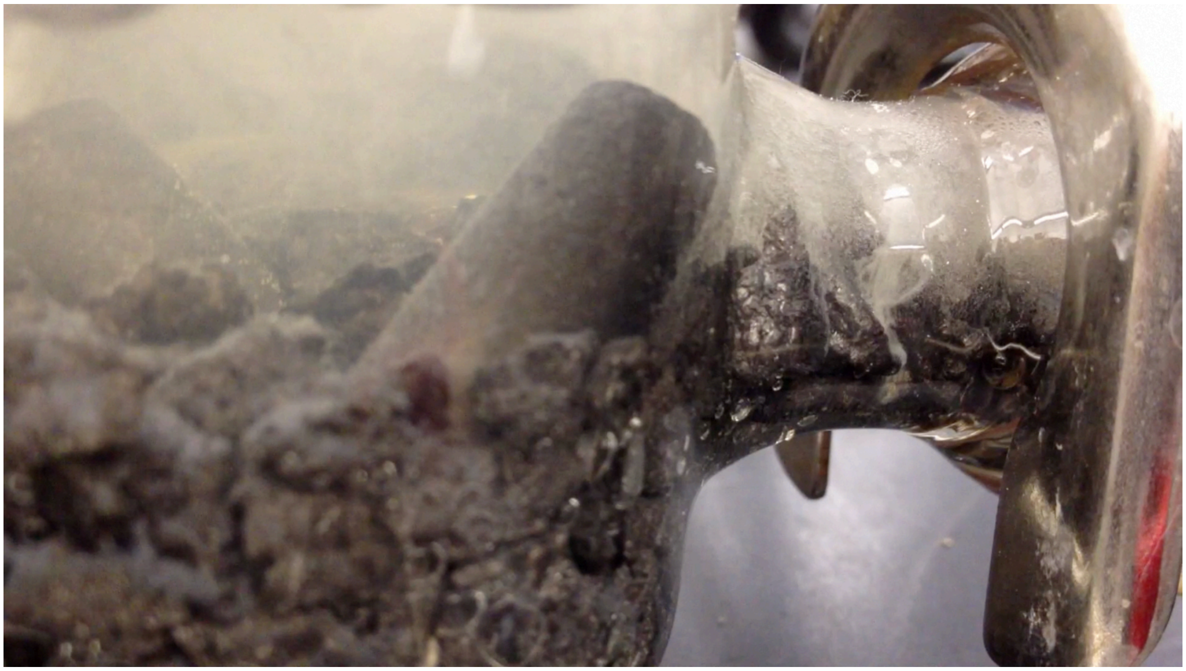

Figure S7. Screenshot of hydrogen gas evolving off of a biocathode. Video screenshot of a biocathode in phosphate buffered media poised at -600 mV vs. SHE.
