# Supplementary material for: Influence of Acidic pH on Hydrogen and Acetate Production by an Electrosynthetic Microbiome
Source: PLoS One. 2014 Oct 15;9(10):e109935. doi: 10.1371/journal.pone.0109935 (PMC4198145; doi:10.1371/journal.pone.0109935)
Supplement: Table S1 — Summarized parameters and maximum productivities for the reactors in this study. Maximum productivities are in mM/day, and g/m2/day is in parenthesis for the rods. Presence of NaBES or NaCl in the catholyte or catholyte and anolyte is noted by C or C & A, respectively. (PDF) [file pone.0109935.s008.pdf]

Reactor	Figure	Electrode	Media	Salt		mV vs. SHE	Hydrogen	Formate	Acetate
1	1A	Granules	Bicarbonate	NaBES	C	-600	330	0.7	4.4
	1B	Granules	Bicarbonate	NaCl	C	-600	422	0.7	11.3
2	S2A	Granules	Bicarbonate	NaBES	C	-600	372	0.3	7
	S2B	Granules	Bicarbonate	NaCl	C	-600	327	0.7	9.4
3	S2C	Granules	Bicarbonate	NaBES	C	-600	328	0.31	3.4
	S2D	Granules	Bicarbonate	NaCl	C	-600	282	1.2	8.2
4	S3A	Granules	Phosphate	NaBES	C	-600	256	0.2	5.2
	2A	Granules	Phosphate	NaBES	C & A	-600	510	3.6	15.8
	4	Granules	Phosphate	NaBES	C & A	-600	1304	1.2	7.7
5	S3B	Granules	Phosphate	NaBES	C	-600	381	0.7	15.3
	2B	Granules	Phosphate	NaBES	C & A	-600	286	4.5	16.3
6	S3C	Granules	Phosphate	NaBES	C	-600	212	0.5	12.6
7	3	Rod	Phosphate	NaCl	C & A	-600	3.9 (0.39)	0.2 (0.46)	1.1 (3.16)
8	3	Rod	Phosphate	NaCl	C & A	-600	18.2 (1.82)	0.9 (2.03)	5.6 (6.28)
9	3	Rod	Phosphate	NaCl	C & A	-600	17.8 (1.78)	0.8 (1.82)	3.6 (10.84)
10	5	Granules	Phosphate	NaBES	C & A	-800	1323	4.54	51.6
	S6A	Granules	Phosphate	NaBES	C & A	-800 to -600	1324	16.9	28.7
	S6B	Granules	Phosphate	NaBES	C & A	-800 to -700	1284	2.9	51.9

Table S1. Summarized parameters and maximum productivities for the reactors in this study. Maximum productivities are in mM/day, and g/m^2/day is in parenthesis for the rods. Presence of NaBES or NaCl in the catholyte or catholyte and anolyte is noted by C or C & A, respectively.
